# Monitoring the reproductive activity in captive bred female ball pythons (*P*. *regius*) by ultrasound evaluation and noninvasive analysis of faecal reproductive hormone (progesterone and 17β-estradiol) metabolites trends

**DOI:** 10.1371/journal.pone.0199377

**Published:** 2018-06-27

**Authors:** Mara Bertocchi, Igor Pelizzone, Enrico Parmigiani, Patrizia Ponzio, Elisabetta Macchi, Federico Righi, Nicola Di Girolamo, Enrico Bigliardi, Laura Denti, Carla Bresciani, Francesco Di Ianni

**Affiliations:** 1 Department of Veterinary Science, Università degli Studi di Parma, Via Del Taglio, Parma, Italy; 2 Ambulatorio Veterinario Belvedere, Reggio Emilia, Italy; 3 Department of Veterinary Science, Università degli Studi di Torino, Via Leonardo da Vinci, Grugliasco (TO), Italy; 4 Tai Wai Small Animal and Exotic Hospital, Lap Wo Building, Tai Wai, Sha Tin, Hong Kong; Auburn University College of Veterinary Medicine, UNITED STATES

## Abstract

The royal python (*Python regius*) is commonly bred in captivity. To have a successful breeding season, accurate monitoring of the reproductive activity is necessary. The use of non-invasive monitoring methods in exotics is important in order to minimize stress. For this purpose ultrasound has been anecdotally used to monitor royal python reproductive activity. However, there is limited information regarding the reproductive cycle of this species. The aim of the present study is to monitor the female reproductive cycle of the royal python using ultrasonography and gonadal steroid metabolite measurements in the faeces. The reproductive activity of one hundred twenty-nine adult female P. regius was examined during two consecutive years. We performed brief scans on non-anaesthetized snakes using a portable ultrasound system and a 10–12 MHz linear array transducer (MyLab™ 30 Gold, Esaote). Ultrasound features, dimension and echogenicity of the reproductive structures were determined. During the second reproductive cycle, the hormonal profiles of 30 animals were also evaluated, with a monthly collection of faecal samples. These samples were classified according to reproductive stage, as identified by ultrasonographic examination, and the mean faecal progesterone and 17β-estradiol levels were calculated using the results from an enzyme-linked immunosorbent assay (ELISA). Progesterone levels increased during the reproductive cycle. Estradiol levels showed greater variability, although they appeared to increase before coupling when compared to the levels between coupling and egg laying. The present study suggests that it is possible to identify different phases in the female royal python reproductive cycle: anovulatory phase, transition, folliculogenesis and embryogenesis. Ultrasound is also useful for identifying follicular regression or slugs. Gonadal steroid metabolite measurements from the faeces could help integrate reproductive information. The use of ultrasonography in addition to the steroid metabolite measurement in the faeces gives an accurate picture of ovarian activity in captive adult female royal pythons.

## Introduction

Reptiles have become increasingly popular pets in recent years, which has led to an increase in captive bred reptiles [1]. The royal python (*Python regius*) is one of the most common reptile bred in captivity, and proper management of its reproduction involves a growing number of veterinary practitioners. These snakes are found in West and Central Africa and are now included in Appendix II of the Convention on International Trade of Endangered Species (C.I.T.E.S., Washington D.C. USA, 1973). In the wild, their breeding season is primarily from mid-September through mid-November, correlating with the rainy season. Royal pythons are oviparous and females lay an average clutch size of 6 eggs, although clutch sizes can range from 1 to 11 [2–4]. In captivity, with proper management of environmental factors, royal python can be bred during any time of the year, including in the winter period [5]. Royal python females reach sexual maturity at a weight of about 1500 g (2 to 2.5 years of age). The ability of a female to produce fertile eggs is linked to the fat reserves of the animal itself [5]. At this point, reproductive behavior and follicular development are stimulated by conditioning, a slight decrease in temperature few months before the expected coupling. In this regard there are several methods. The room temperature can be gradually reduced from 31°C to 28°C during the day and from 29°C to 24°C at night, lowering it by about 2°C every two days, leaving a point at a higher temperature available to the animal (29–31°C) [6,7]. During conditioning, some authors suggest not only modifying the temperature but also the photoperiod [7]. The ovaries of adult females present follicles throughout the year, but these are generally small in size. Due to increased body weight and environmental factors, follicles develop during the breeding season and will be reabsorbed in case of non-fertilization [4,5]. Upon ultrasound evaluation, follicles appear initially anechoic. As vitellogenesis progresses, follicular diameter increases together with echogenicity, resulting in a typical soft tissue echogenicity [4,8]. At this point, follicles assume a target-like appearance [4]. The shape of follicles also tends to vary, changing from round to oval. From a clinical perspective, ovulation is associated with mid-body swelling. However, there are no published studies that claim that vitellogenesis and mid-body swelling coincide [4]. After the pre-egg laying shed, the female lays the eggs in an average of 30 days [5]. Eggs can be left to the mother or artificially incubated. In case of maternal incubation, the female must not be disturbed and the temperature and humidity conditions must be checked. The mother remains rolled around the eggs until these will start to open, tightening or loosening her coils depending on the eggs temperature [5]. Often in professional breeding, eggs are artificially incubated. Indeed, during hatching, royal pythons cease to feed, so the time needed for recovering the appropriate body weight for reproduction is greater for these females, compared with females whose eggs have been artificially incubated [5]. Monitoring the female reproductive cycle and controlling gestation and deposition are critical for successful breeding [9,10]. Therefore, it is necessary to have reliable techniques for monitoring the gonadal function in each animal [11]. In recent years, several non-invasive methods for monitoring ovarian function have been developed to reduce stress due to handling [12–14]. Palpation is commonly used to assess the reproductive status of reptiles, and though it can be informative, it is also risky, potentially traumatic, and can be complicated to perform on large specimens [15]. Radiography can identify and localize soft tissues, but they are not well-defined [16]. It also requires a motionless patient, which often involves sedation. Endoscopy and laparoscopy allow direct visualization of internal organs but are invasive procedures requiring general anesthesia. Conversely, ultrasound allows a good assessment of the internal structures, including those not calcified, and most of the time can be performed with manual restraint [17,18]. Currently, ultrasonography is the most common techniques used in veterinary medicine for evaluating reproductive activity in both mammals and reptiles [19–30]. Ultrasound evaluation of the female gonads in reptiles has been extensively studied in chelonians [31–35], as well as in lizards [36–39] and in some species of snakes [15,16,40,41]. Sonographic examination of the female gonads presents some interspecific differences. In snakes and lizards, inactive ovaries appear anechoic and small; however, in chelonians, they appear hyperechoic [16,23,24,36]. Ovarian follicles are described as structures without the characteristic concentric appearance of calcified eggs. The latter may show a shell that is echogenic depending on the amount of calcification. Some authors have also shown ultrasound embryonic structures and movements in ovoviviparous species [16,18,23–25,36,42]. Similarities have been found in the morphology and topography of the gonads in some lizards (such as green iguanas) and snakes, but there is still a need for further research on each species due to the large number of snake species, the lack of a description of the sonographic appearance of ovarian structures during the reproductive cycle, and a lack of information on the existence of certain phases during the cycle. Knowledge of the appearance upon ultrasound of normal reproductive structures in female snakes is critical for early detection of possible diseases, such as ovarian retention and tumors [18,43]. Ultrasound appearance of reproductive organs in reticulated pythons (*Python reticulatus*) allowed recognition follicles in various stages of development, as well as calcified eggs [44]. In this species, ultrasound is a potential diagnostic method for highlighting pathological conditions and monitoring healthy animals to identify the optimum time for mating [44]. Ultrasonography has also been used in rattlesnakes (*Crotalus atrox*) to assess changes in reproductive structures associated to reproductive parameters such as hormonal profiles and behavioural manifestations [15,45]. To fully describe the reproductive process in a certain species, sex steroid levels during the reproductive cycle should be also evaluated [46–48]. Steroidal hormones regulate numerous physiological processes in vertebrates, including growth and reproduction, and the role of these hormones in reproduction has been widely studied [46,47,49–51]. Sex steroids are important regulators of the physiological and behavioural components of reproduction in reptiles. In some species, oestrogens, such as 17β-estradiol, stimulate sexual behaviour and vitellogenesis in females, while androgens (testosterone) stimulate sexual behaviour and spermatogenesis in males [47]. Progesterone seems to be involved in maintaining pregnancy, with high serum levels in species such as *Thamnophis elegans* and *Vipera aspis* [52–55]. Assessing sex steroids provides essential information for managing reproduction and breeding in animals. Hormonal variation is related to events such as sexual maturity, gestation and transition between phases of the reproductive cycle [48]. Although detecting concentrations of sex steroids in the serum is the most direct method of assessing hormone levels, obtaining blood samples could be difficult due to the technical complexity of the sampling. It is also stressful for the animal, particularly in exotic and wild species. Therefore, non-invasive methods of detection, such as monitoring hormone levels in faeces, urine and saliva, have been studied in many species [56–74]. Non-invasive endocrine monitoring by collecting faecal material, allows long-term repeatable sampling at frequent intervals [48,61,75,76]. Direct comparison of steroid serum concentrations and metabolites in the excrement can be difficult; thus, the variability of the methodology must be considered. However, correlations between serum hormone levels and faecal metabolites have been demonstrated in several species [11,48,77–79]. Sex steroids were evaluated in certain reptile species, primarily with plasma and serum samples [50,54,80–86]. The most common technique is the radioimmunoassay (RIA, *Radio Immuno Assay*) [50,81,83,85,87,88]. However, the enzyme immunoassay (EIA) eliminates the use of radioactive material and has been used in several species [11,76,89]. There is little in the literature on royal python reproductive activity, and to date research on the assessment of sex hormones in this species have not been reported. Therefore, the aim of this study is to monitor the reproductive cycle of captive female royal pythons using ultrasonography and gonadal steroid faecal metabolite analysis.

## Materials and methods

The protocol of the study was approved by the “Organismo preposto al benessere degli animali (OPBA)”, ethics committee of the University of Parma (PROT.N. 24/OPBA/2014). All ultrasound sessions were performed on unsedated animals with minimal manual restraint and all efforts were made to minimize stress.

### Breeding structure

The study was conducted at the professional breeding facility, “MC Snakes” (Florence, Italy). The structure houses more than 1000 adult females and approximately 100 adult males, with approximately 5000 births per year. Females being used for reproduction are all reared in standardized housing, with standard handling and environmental conditions. Each female is individually housed in a rack measuring 80x40x20 cm, with substrate comprised of dedusted beech chips and a water bowl. The warm side of the enclosure is maintained at 31°C and the cool side at 28°C. To stimulate reproduction, the enclosures are conditioned by gradually lowering the highest temperature to 25°C (1°C per day) and so maintained for three months (October, November and December), which is identified as the cooling period (CP). After the CP, the temperature is gradually raised back to 31°C (1°C per day). A probed thermostat located in the rack allows the temperature to be monitored. The humidity is kept between 50% and 60% and the L/D cycle is set at 12:12, also during the CP. After deposition, eggs are artificially incubated.

### Animals

One hundred twenty-nine female royal pythons were included in this study. All were two year old adults, weighing 1800±200 g and with a length of 130±10 cm. The animals, all born in captivity, were fed every four days with captive bred frozen/thawed rats that weighed between 100 and 200 g. Food was offered up to the time of mating, but food intake was variable. All animals were subjected to ultrasound monitoring, and a random subgroup of 30 snakes was also evaluated for the hormonal profile, by collecting faecal samples monthly.

### Management data collection

Animals were monitored over two consecutive breeding seasons in 2015 and 2016, and checked at least daily. Data collected included: medical history, conditioning, coupling, rack temperature, pre-egg shed, number of fertile eggs and number of slugs laid. Coupling occurred when at least one follicle reached 10 mm in diameter, and at this time, a male was housed with the female for 24 hours and monitored.

### Ultrasound assessment

After the temperature was restored to 31°C at the end of the CP, ultrasound monitoring was performed daily until coupling. Animals were monitored at this frequency until egg deposition and then over the next 30 days. From then on, animals were monitored by ultrasound every two weeks until the end of the next CP. Ultrasounds were performed on unsedated animals, and the animals were maintained in sternal recumbency on a table and lifted to facilitate evaluation of the ventral side. After applying ultrasound gel, brief scans (approximately 5 minutes) were performed to minimize the stress to the animals. The probe was orientated transversely, and lateral and ventral scans of the medium and caudal third of the python’s body were obtained in accordance with Hochleitner and Hochleitner [24] and Schilliger [18]. We used the portable ultrasound, MyLabTM 30 Gold (Esaote, Genoa, Italy), equipped with a linear probe 533 set at 10–12 MHz. Depth and contrast were adjusted to optimize structure display. The following characteristics of the reproductive cycle were highlighted based on their sonographic appearance:

- presence of follicles, their size, and echogenicity
- presence of eggs, their size, and echogenicity
- embryonic structures and their viability (by Power Doppler)

The ultrasound session data were reported in tables for each python, including the date, the largest follicular diameter in mm, the sonographic appearance of the observed structures and any additional notes such as skin shedding, mating and egg laying. Using an Excel spreadsheet, time intervals (in days) were calculated for the most significant reproductive events: the beginning of ovarian follicular development after the CP, introduction of the male and mating, skin shedding, and the deposition of fertile eggs and/or slugs. The follicular growth rate (mm/day) was evaluated, considering the day that the eggs were laid as the zero point, we measured the period between deposition and coupling, with an intermediate evaluation at 5 mm in diameter, and the time interval between coupling and deposition.

### Faecal sample collection

During all the phases of the second reproductive cycle (ie from deposition to CP end, from the CP end to coupling, and from coupling to the eggs laying), a randomly extracted subgroup of 30 animals was used to collect monthly faecal samples to assess the presence of sex steroid faecal metabolites (SSFM). Available samples were collected fresh (within a few hours after defecation), placed in plastic bags and stored at -20°C until analysis. Considering the particular physiology of the species, it was not possible to have a sample for each animal at each stage of the reproductive cycle, therefore a pool of faecal samples for each stage was collected (for a total of 88 samples collected during the reproductive cycle). Each of these samples was valid as associated with a particular stage of the reproductive cycle by performing an ultrasound scan on the same day as the sample collection. Each sample was labelled with the animal's code and date of collection. The hormone assay was performed within six months of the sample collection.

### Steroid hormone measurement

To extract the steroids, we used the methanol-based procedure described by Palme et al. [90] with slight modifications. Briefly, the faeces were lyophilized, weighed, and crushed, and then two aliquots of the sample (0.25 g each) were placed into extraction tubes, sealed with a Teflon cap and stored at −20°C. Each aliquot was thoroughly mixed for 30 min using a multivortex with one mL of 80% methanol (Sigma Aldrich, St. Louis, MO, USA). The suspension was then centrifuged at 500 g for 20 min and the supernatant was recovered. An aliquot (0.5 mL) of the supernatant was transferred into a new vial and evaporated at 50°C for 14 h. After evaporation, the dried extracts were stored at room temperature in dark boxes for 15 days and then kept at −80°C until they were assayed. One day before the SSFM analyses, the dried extracts were re-diluted in 0.5 mL of 80% methanol. An aliquot of the extract was diluted to 1:10 in the assay buffer (Arbor Assay^®^, Ann Arbor, MI, USA). The mixture was then vortexed and left to rest for 5 min twice to ensure complete steroid solubility. Faecal immunoreactive progestogen (FPM) and oestrogen (FEM) metabolite concentrations were determined using two multi-species progesterone and estradiol enzyme immunoassay kits (K025-H5, K030-H5; Arbor Assay, DetectX®, Ann Arbor, Michigan, USA) to determine steroids on different biological matrices such as faeces and urine. All analyses were repeated twice. The concentration of FPM and FEM was expressed as ng/g and pg/g of faeces dry matter.

### Validation of the test

#### Repeatability and reproducibility

The inter- and intra-assay coefficients of variation were < 10% for all assays. All faecal samples were analysed at multiple dilutions (1:4, 1:8, 1:16 and 1:32) and all regression slopes were parallel to the standard curve (r2 = 0.987). The Recovery of the P4 and E2 (antibodies) were determined by adding increasing amounts of the analyte to six different faecal samples containing different amounts of endogenous analyte (low, medium, and high amounts). Each sample was assayed and analyte concentrations of the samples were calculated from the standard curve. The percentage recoveries were determined by comparing expected and measured values of the samples. The recovery rates of P4 and E2 (antibodies) added to the dried faeces were 94% and 72%, respectively.

#### Sensitivity and cross-reactivity

Sensitivity and specificity of the kit have been validated by serial dilutions of the sample, previously extracted, so as to verify a linear correspondence between dilution and result [91]. The P4 and E2 test sensitivities were determined by measuring the least amount of hormone standard consistently distinguishable from the zero concentration of the standard and were 0.96 ng/g faeces and 19.4 pg/g faeces, respectively. Per the manufacturer, the P4 antibody used to quantify faecal P cross-reacts 100% with progesterone, 172% with 3ß-hydroxy-progesterone, 2.7% with 11ß-hydroxy-progesterone, 7% with 5a-dihydroprogesterone, <0.1% with corticosterone and 5.9% with pregnenolone. The E2 antibody cross-reacts 100% with 17β-estradiol and 0.73% with oestrone.

#### Clinical validation

Ideally, faecal steroid profiles should be validated by evaluating the plasma hormones [92]. However, this is not always possible for non-conventional or wild animals, which are particularly sensitive to stress, as this is an invasive method of monitoring and not repeatable at regular and frequent intervals in healthy animals [93–95]. Clinical validation was performed on the animals’ reproductive behaviours including acceptance of the male, mating and depositions, which were observed and recorded daily. The animal’s reproductive status was clinically identified through ultrasonography. The follicular stages of development and degrees of calcification were observed from the ultrasound recording, and an absence of follicles indicates reproductive inactivity [4,18,44].

(The described protocol has been deposited in protocols.io, with the following DOI: http://dx.doi.org/10.17504/protocols.io.kttcwnn)

### Statistical analyses

Whereas ultrasound examination is an operator-dependent technique, for a subgroup of animals (n = 15) we have evaluated intraobserver variability (IAOV) and interobserver variability (IEOV) [96]. IAOV and IEOV were measured and expressed as coefficient of variation (SD/mean value) between measurement. In particular, IAOV was calculated on two recordings made by a single examiner during an ultrasound session. Instead, the IEOV was calculated on data measured both together by an independent second examiner during the same ultrasound session [96]. Both parameters were expressed as percentage, according to Wasmeier et al. [96] and repeated for the reproductive cycle phases (ie from deposition to CP end, from the CP end to coupling, and from coupling to the eggs laying). The statistical analysis was performed using the statistical software package SPSS for Windows (IBM SPSS Statistics for Windows, Version 21.0. Armonk, NY: IBM Corp). The analysis of variance was conducted through the Univariate Procedure of the General Linear Model (GLM), using the reproductive phases as fixed effect; Bonferroni Post Hoc Test was performed to determine the significance, which was set at P≤0.05. Variabilities were compared using the T-test.

## Results

### Ultrasound assessment

**Fig 1:**
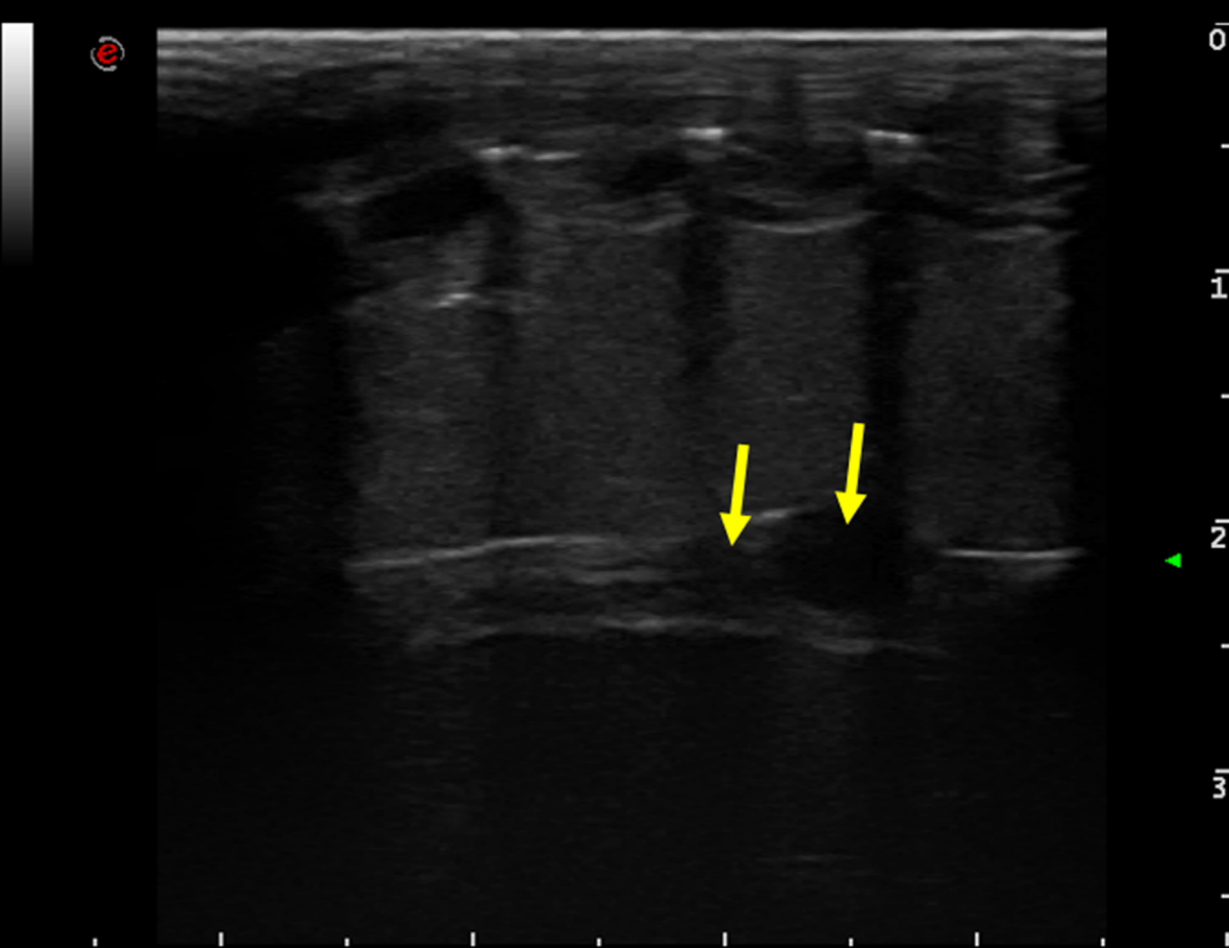
Long axis ovary scan. With a ventrolateral approach, few follicles (arrows) in the ovarian stroma are highlighted. The follicles appear anechoic and round. The large bars on the right side and lower border indicate a distance of 1 cm.

In our study, IAOV resulted similar (P>0.05) to IEOVin the first reproductive phase (1.61 vs 1.88%), in the second (0.89 vs 0.85%) and in the third one (11.05 vs 17.32%). Both variabilities were low in CP end–coupling and coupling-deposition (IAOV: 1.29 and 0.89%; IEOV: 1.89 and 0.85%, respectively) and high in deposition-CP end (IAOV: 11.05%; IEOV: 17.32%). Before the CP, anechoic ovarian follicles that are small (less than 5 mm in diameter) and round in shape are recognizable by ultrasound (Fig 1).

**Fig 2:**
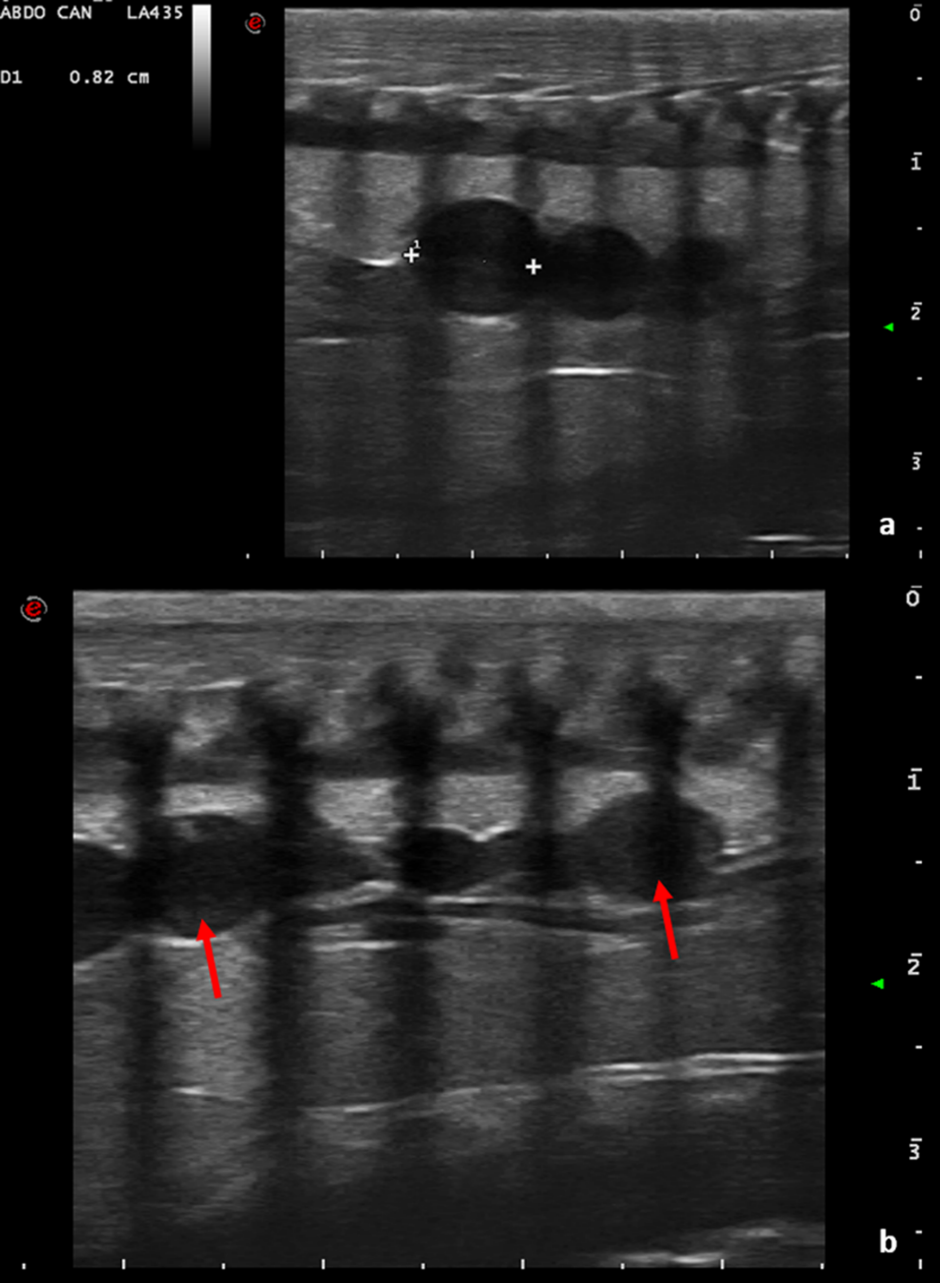
Follicles chain-aligned. (a) With a ventrolateral approach, follicles chain-aligned are highlighted. The follicles appear anechoic and round. The large bars on the right side and lower border indicate a distance of 1 cm. (b) Follicles chain-arranged. The follicle size varies; however, the largest are between 5 and 10 mm in diameter (arrows).

Once the CP is complete, more follicles can be observed by ultrasonography. These structures vary between 5 and 10 mm in diameter and often appear with “chain” alignment (Fig 2).

**Fig 3:**
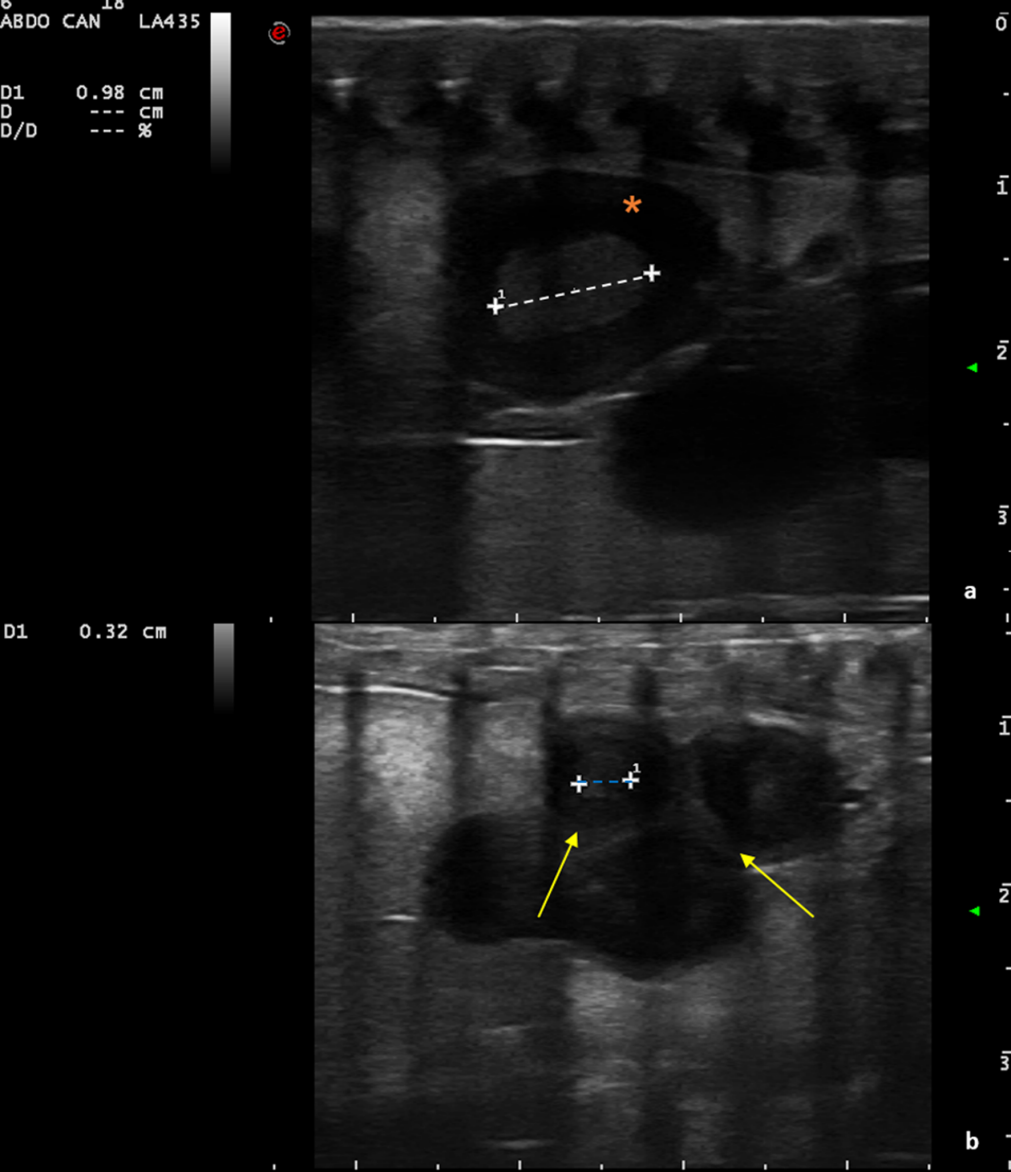
Follicles undergoing villogenesis (dotted lines) with some having an anechoic peripheral area. (a) (asterisk), and others with more pronounced external echogenicity (b) (arrows). The large bars on the right side and the lower margin indicate a distance of 1 cm.

**Fig 4:**
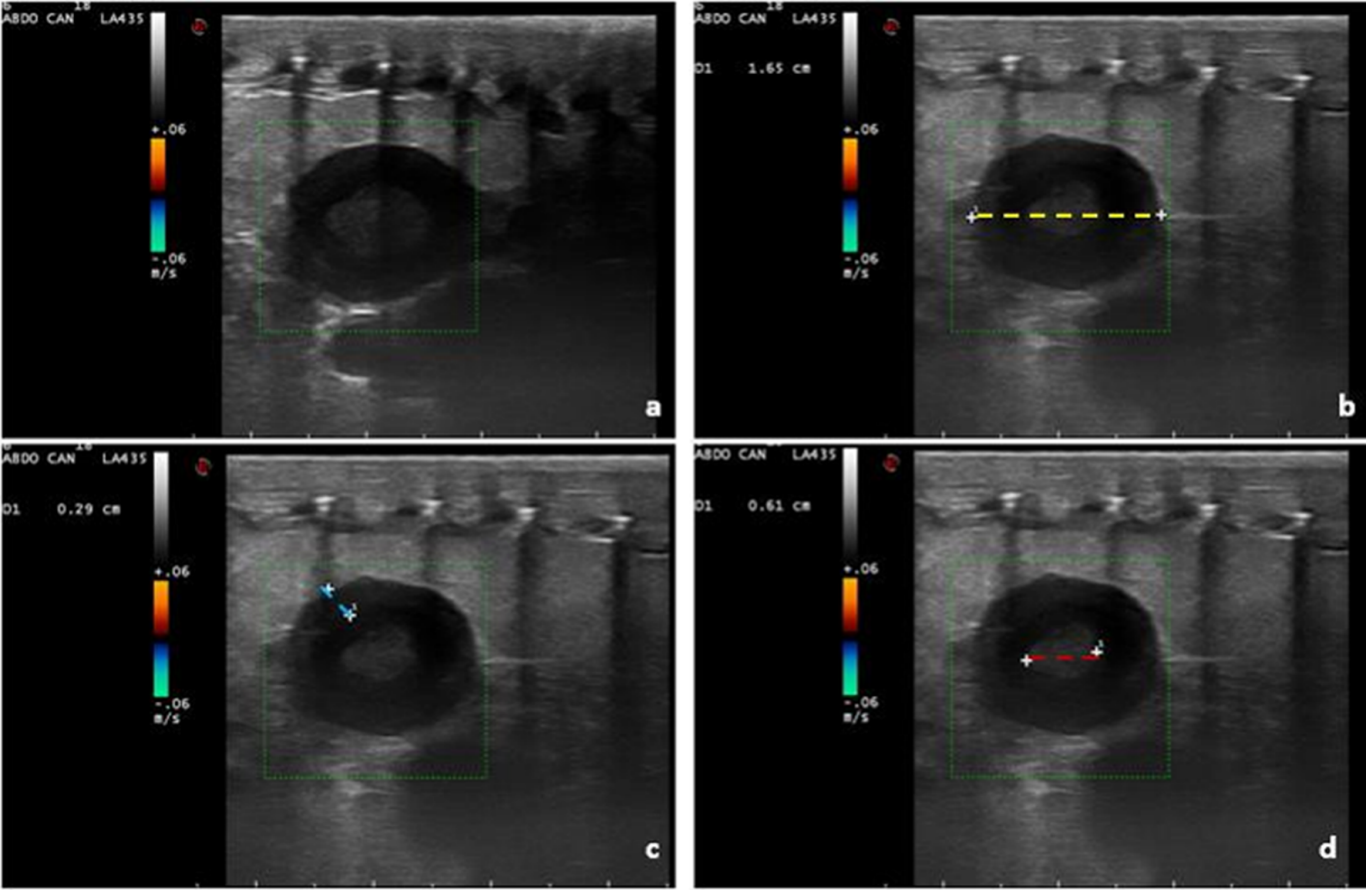
Follicle evaluation. Lack of vascularization (a); follicular diameter (dotted yellow line) (b); peripheral area with higher echogenicity (blue dotted line) (c); central hyperechoic area—yolk (red dotted line) (d).

Initially, follicles are round and anechoic, and then tend to show a greater peripheral echogenicity with the increase in size. When the female has accepted the male for mating, at least 1 follicle of 10 mm in diameter is visible on the ultrasound. Follicles increase in diameter and vary in echogenicity. Initially, some follicles appeared uniformly anechoic, while others had an increased peripheral echogenicity, leading to an increased central echogenicity. In some cases, this resulted in hepatomegaly. The peripheral portion of the follicles also became gradually hyperechoic, giving the follicle a target-shaped appearance (Figs 3 and 4).

**Fig 5:**
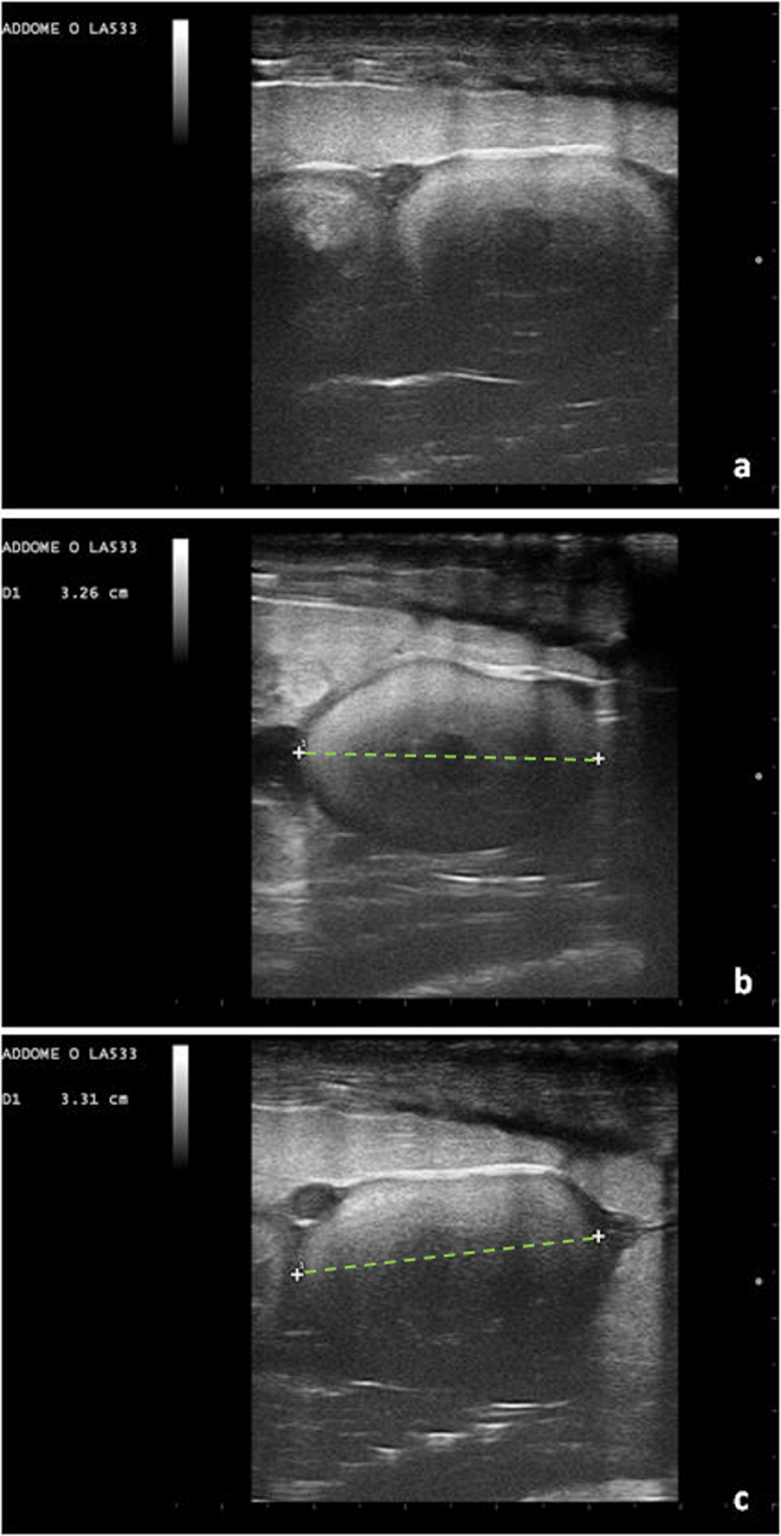
Follicular diameter >30 Mm (dotted lines). (b, c); oval shape (a, b, c). A less echogenic central area is present. The calcified shell is not yet noticeable.

As the follicle develops, the oval shape becomes more evident, but still has irregular echogenicity (Fig 5).

**Fig 6:**
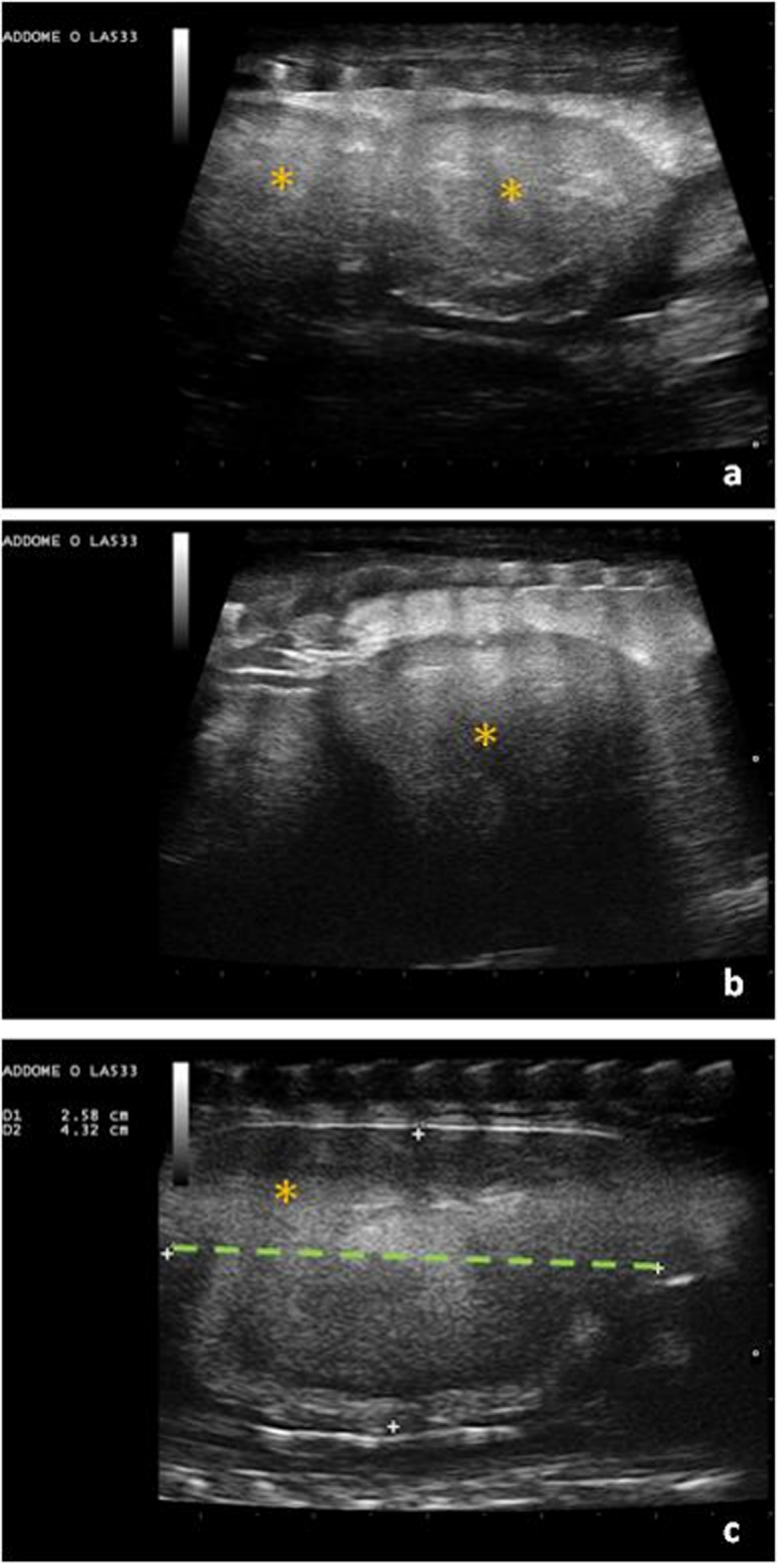
A more advanced stage of development. Structures (asterisks) greater than 30 mm in diameter (dotted line), in a more advanced stage of development. (a, b, c).

Then, the appearance of fertile eggs becomes more homogeneous (Fig 6).

**Fig 7:**
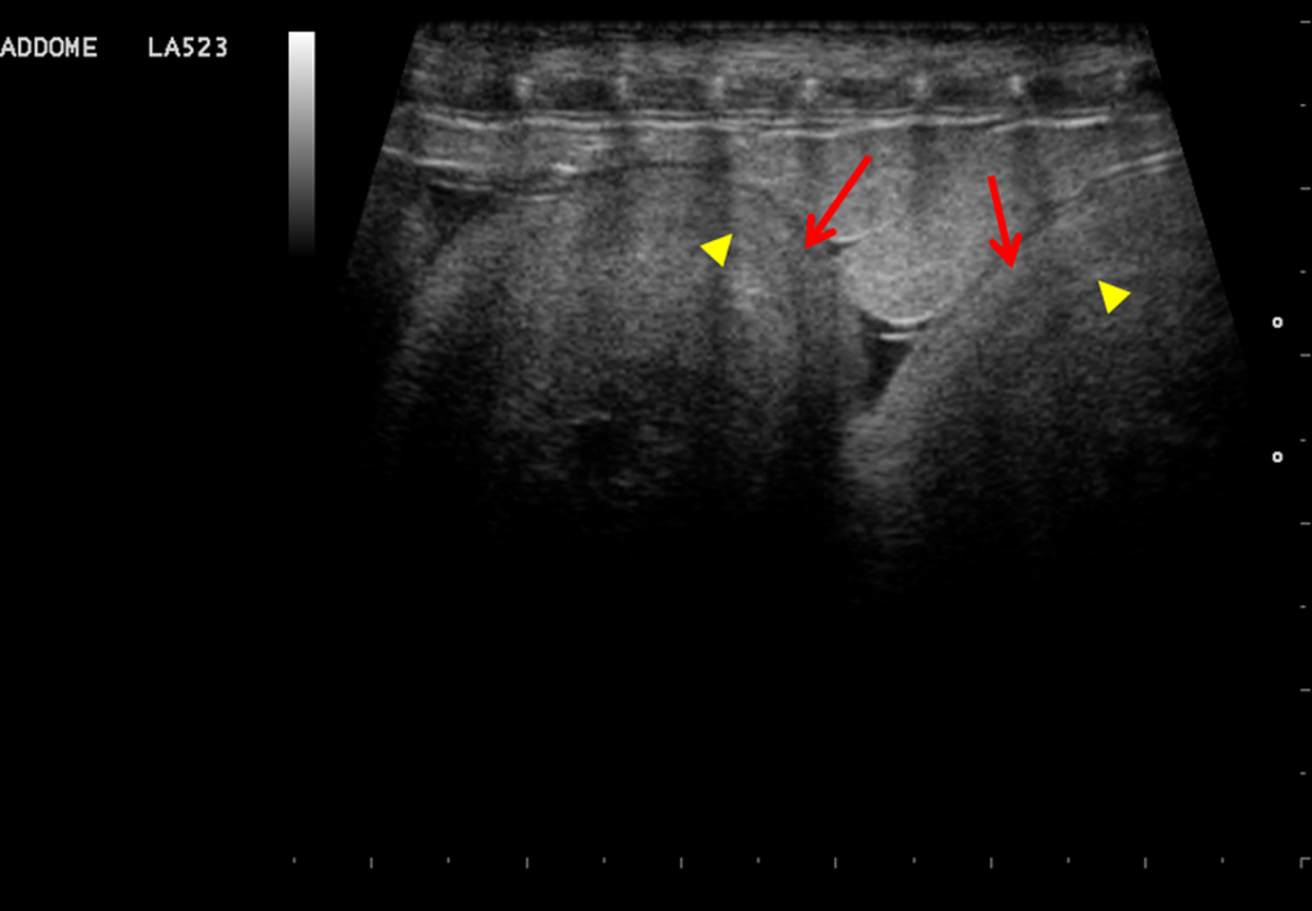
Eggs with calcified shell (arrows) and inner wall (arrowheads).

As they develop, eggs are characterized by a double peripheral layer, consisting of a more externally calcified outer shell and an inner membrane (Fig 7).

**Fig 8:**
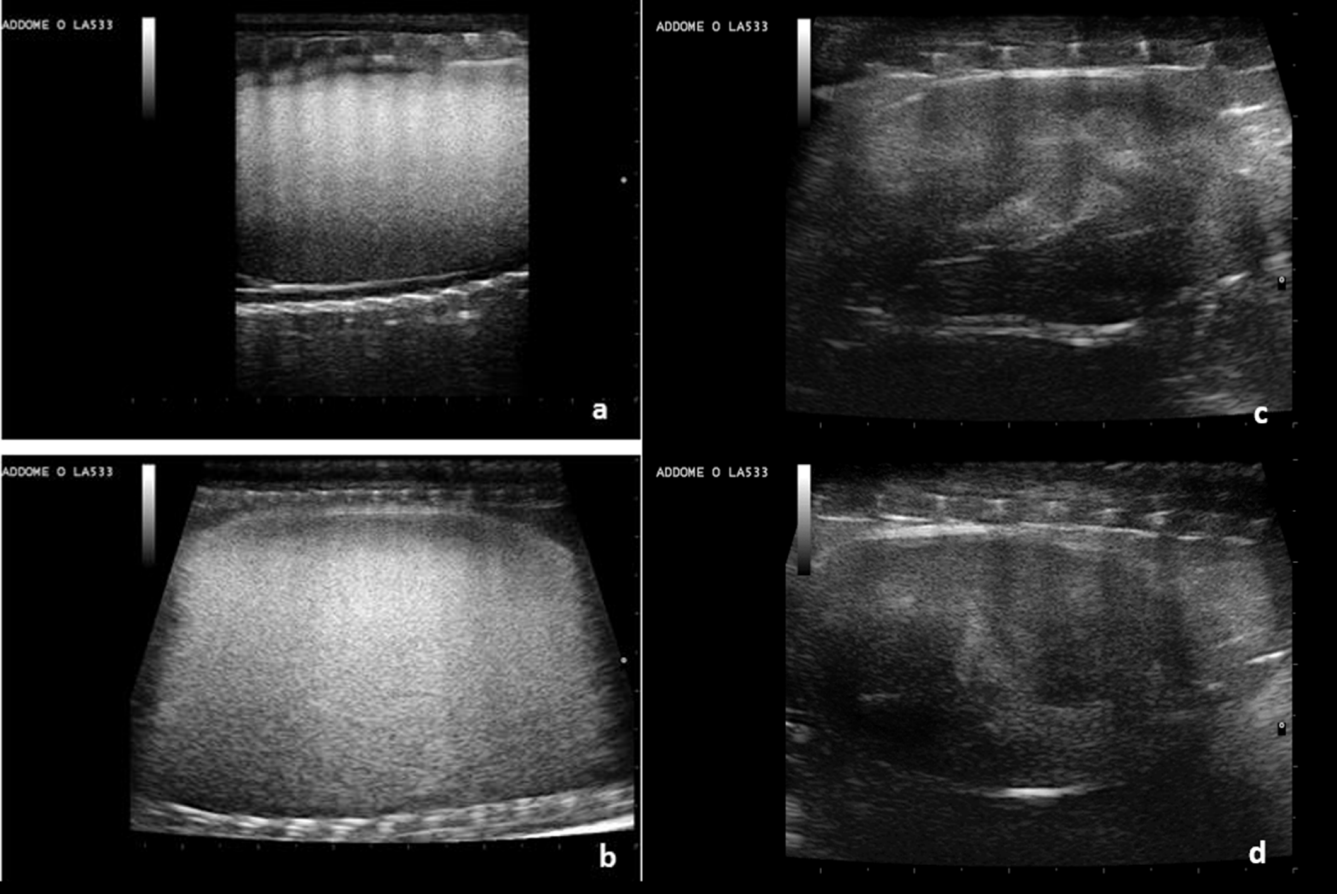
Three weeks before deposition. Some eggs are similar in size and appearance, i.e., oval-shaped and uniformly hyperechoic (a, b); Two eggs show irregular echogenicity (c, d); The female later deposited 7 fertile eggs and two slugs.

Structures in some animals appeared different and irregular on the ultrasound at three weeks before deposition. (Fig 8).

**Fig 9:**
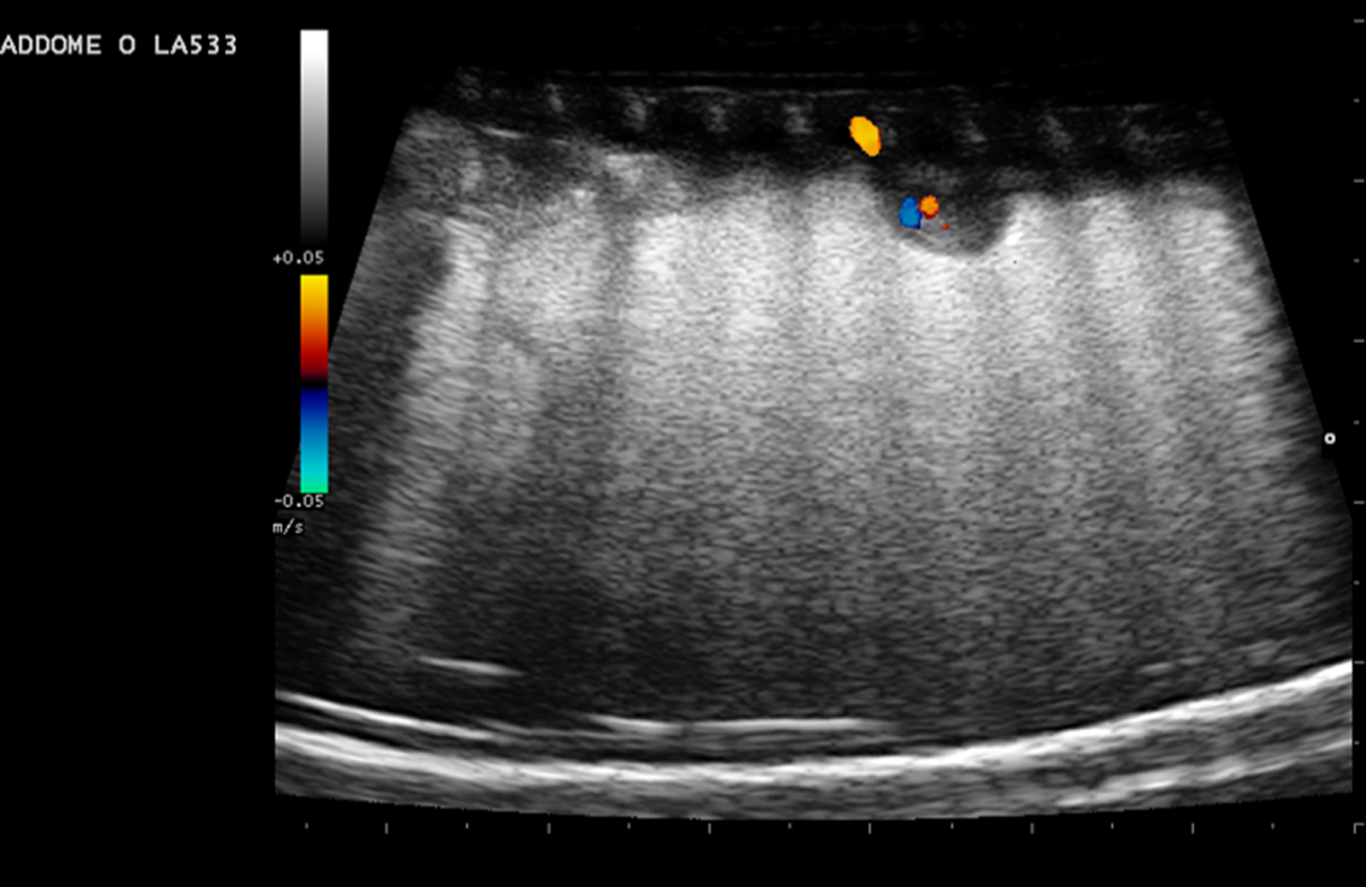
Eggs two weeks before deposition. The embryonic vesicle can be seen, and blood flow is highlighted by colour doppler.

**Fig 10:**
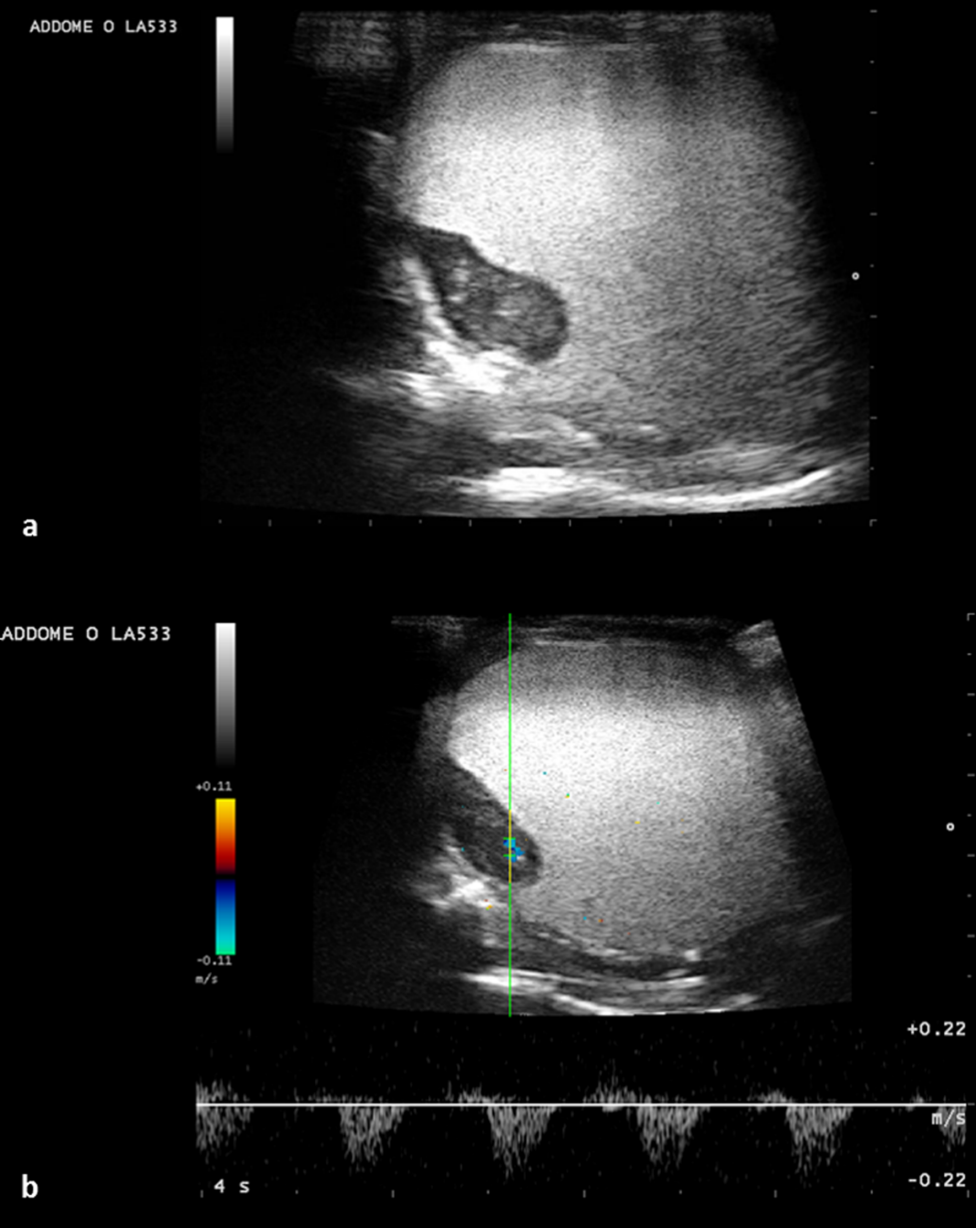
One day before deposition. **evaluation of initial cardiac activity: Image from a B-mode video.** (a) with Pulsed-Wave (PW) Doppler (b).

The embryonic vesicle can be seen on the sonogram approximately two weeks before egg deposition. By using colour Doppler, vitality, embryo vascularization and early heart activity could be assessed. Placing a sample volume over the embryo heart, systolic (0.22 m/s) and diastolic (0.07m/s) peak velocity and heart frequency were evaluated. Thus, as the deposition time approached, embryonic development, vascularization and viability were monitored (Figs 9 and 10, S1 and S2 Videos).

**Fig 11:**
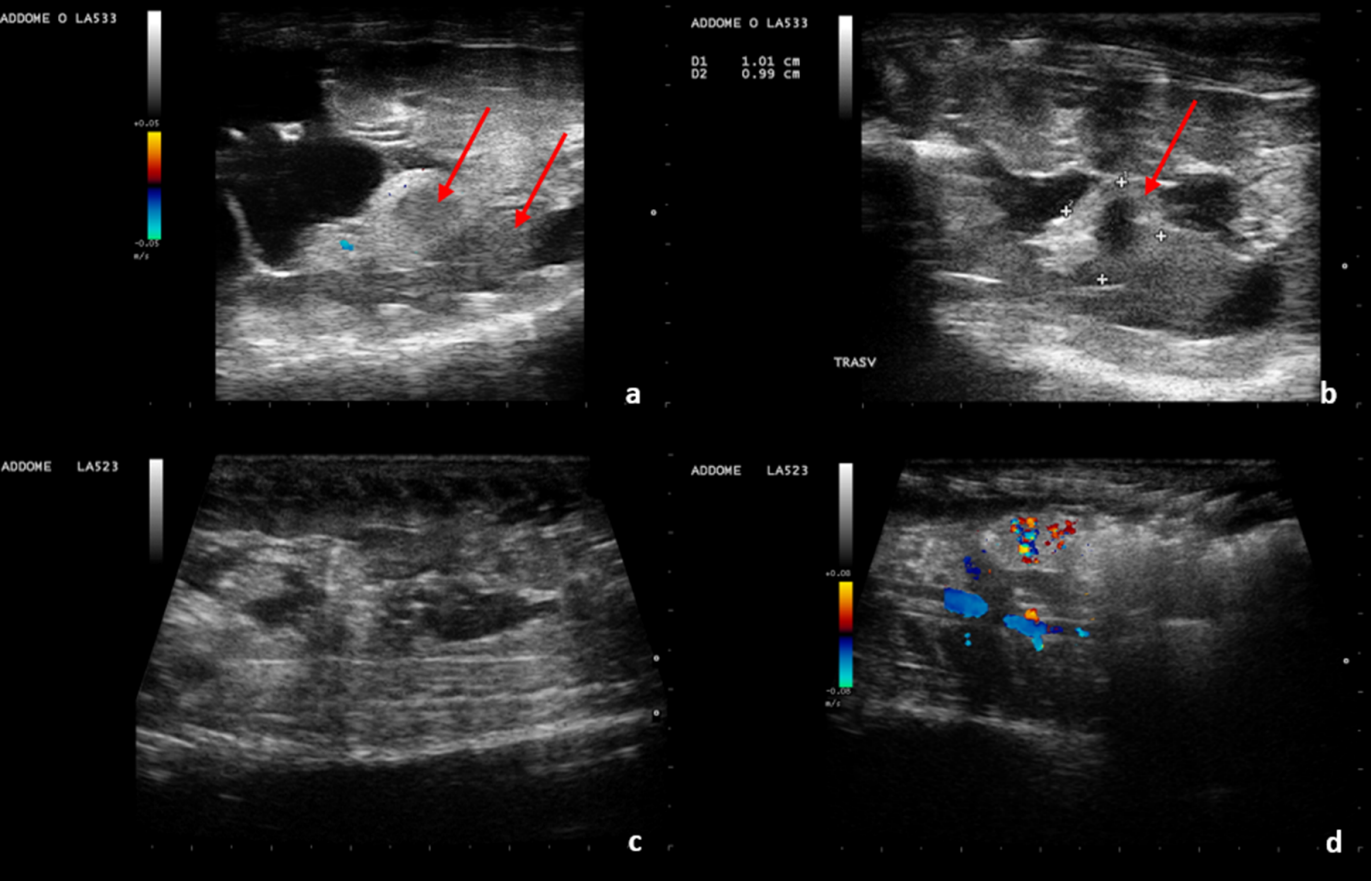
Few hours post-deposition. **Ultrasound Evaluation.** (a, b) A few hours after deposition some rounded structures characterized by peripheral hyperechogenicity and central hypoechogenicity are shown (arrows). Some anechoic fluid is also present. (c, d) One day after deposition, these structures are no longer clearly recognizable.

Evaluations after egg deposition were also performed. On the day of deposition a few rounded structures of approximately 1 cm in diameter with peripheral hyperechogenicity and central hypoechogenicity are shown. These structures cannot be seen on the day after deposition (Fig 11).

**Fig 12:**
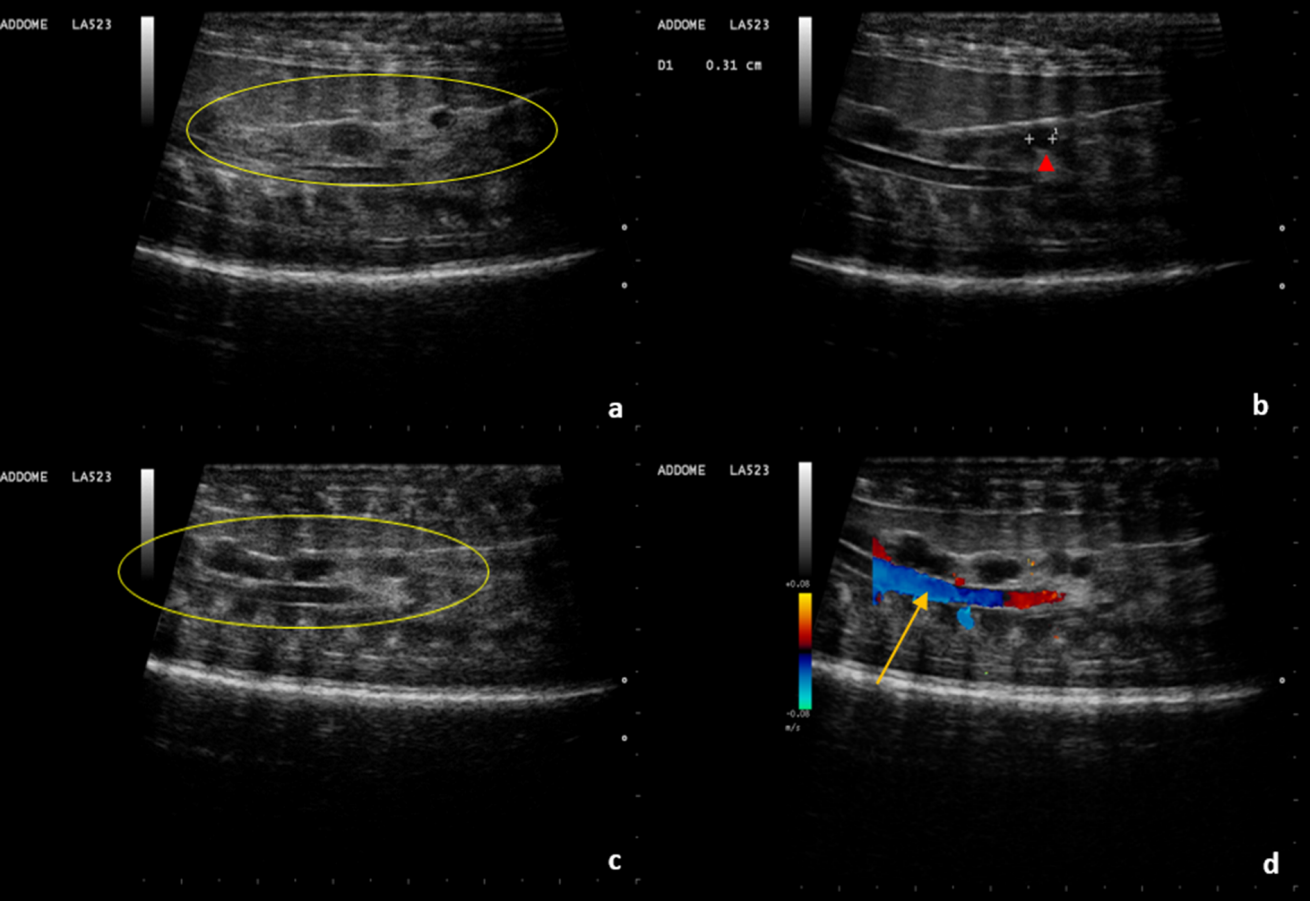
One month post-deposition ultrasound evaluation. (a, b, c) In the ovarian stroma (yellow oval), small roundish anechoic structures less than 5 mm in diameter are highlighted (arrowhead). (d) Aorta (arrow).

A month after deposition, a few anechoic round structures less than 5 mm in diameter corresponding to the ovaries, were recognizable (Fig 12).

Cases of follicular regression were observed. Follicles over 10 mm in diameter regressing to an appearance characteristic of the previous stages of development were observed, with a decrease in diameter and changes in echogenicity.

#### Follicular growth rate and mean length of time

**Fig 13:**
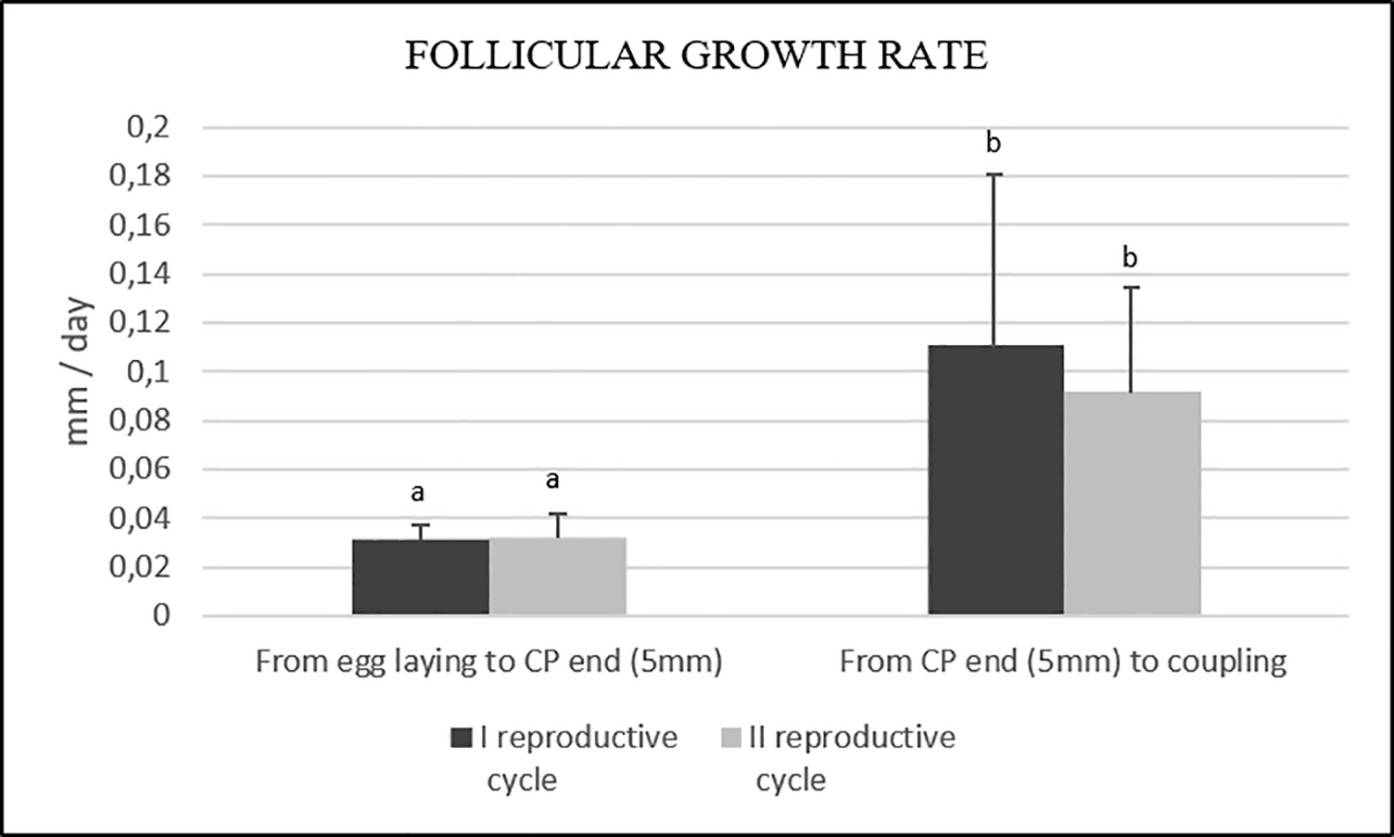
Average follicular growth rate (MM/Day) during the first and second reproductive cycles. Error bars represent the standard deviation. a, b: P ≤0.05.

During the first reproductive cycle, there was a statistically significant difference between the reproductive quiescence and the next period between the rise of the ambient temperature at 31°C (end of CP and at least one follicle of 5 mm in diameter) and coupling. The follicular growth rates were 0.031±0.006 mm/day and 0.110±0.071 mm/day (P<0.01), respectively. There were no significant differences between the two reproductive cycles (P>0.05) (Fig 13).

**Fig 14:**
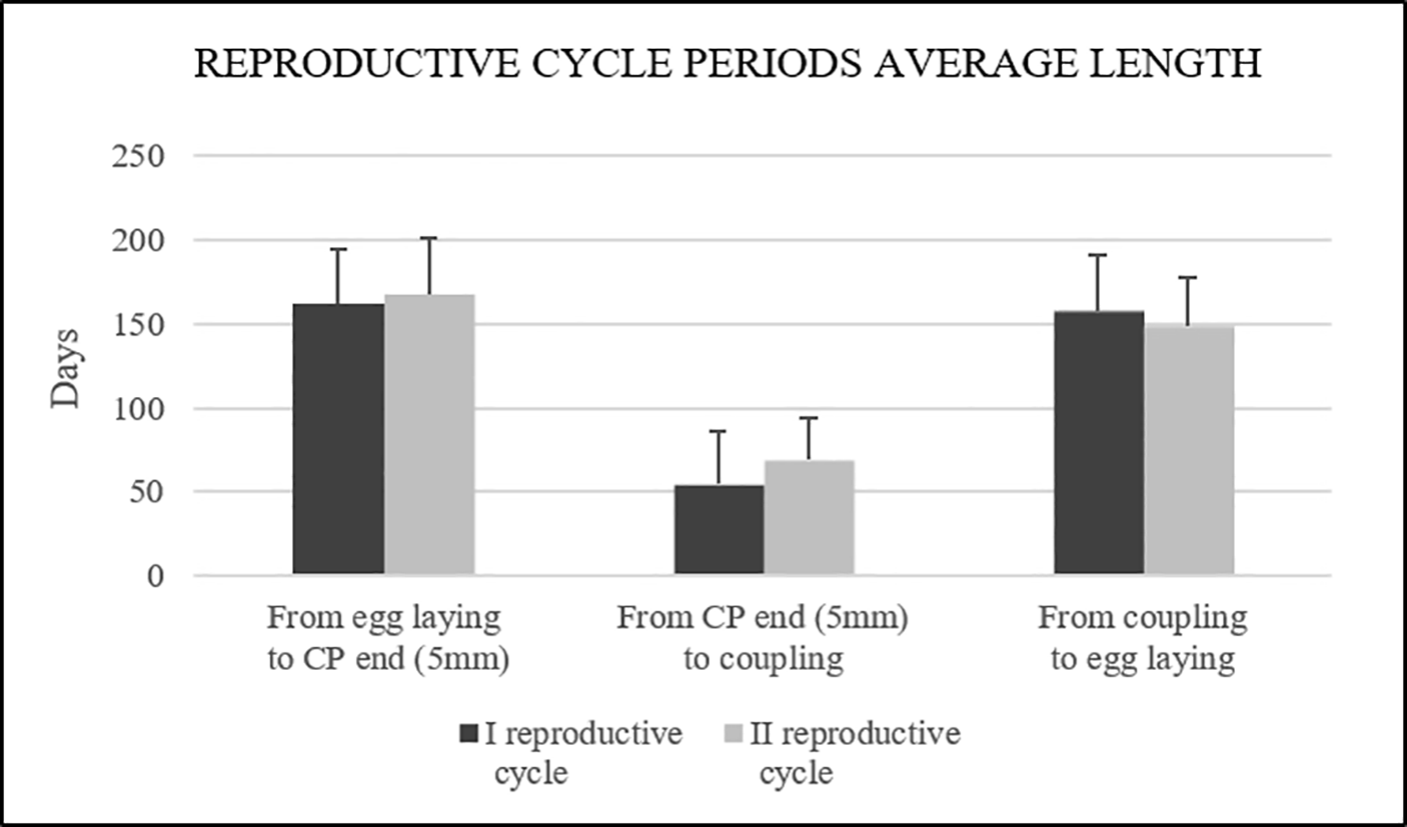
Phases average length in days during the first and second reproductive cycles. Error bars represent the standard deviation.

The average length of time required to attain at least one follicle of 5 mm in diameter after egg laying was 166.23±32.92 days, and to obtain a diameter between 5 and 10 mm was 62.79±27.50 days. The average time interval between coupling and deposition was 157.33±34.08 days (Fig 14). There were no significant differences between the two reproductive cycles (P>0.05).

The pre-egg laying shed occurred 28.8±4.4 days before deposition, and it was possible to evaluate the embryonic vesicle 12.1±2.3 days before deposition. There were no statistically significant differences between the two reproductive cycles (P>0.05).

#### Egg and slug counts

The mean number of eggs laid during the first reproductive cycle was 6.3±2.3, and the mean number of slugs was 1.5±0.6. There were no statistically significant differences in the mean number of eggs laid during the second reproductive cycle, 5.8±2.7 (P>0.05). In contrast, the average number of slugs increased to 3.8±2.4, which was significantly different (P <0.01)

### Evaluation of hormonal trends

As reported in Table 1, the progesterone level increased during the reproductive cycle.

**Table 1:** Mean FPM (ng/g) and mean FEM (pg/g) levels during the reproductive cycle. The number of fecal samples obtained for each phase of the reproductive cycle is shown in brackets. The analysis of variance was conducted through the Univariate Procedure of the General Linear Model (GLM), using the reproductive phases as fixed effect; Bonferroni Post Hoc Test was performed. Mean value ± SE is reported. A, B: P ≤0.05.

	From egg laying to CP end (5mm) (n = 14)	From CP end (5mm) to coupling (n = 8)	From coupling to egg laying (n = 66)	P

**FPM (ng/g)**	38,71±4.52 a	66,75±4.15 ab	99,54±6.61 b	<0,001
**FEM (pg/g)**	82,55±15.90	103,57±8.39	75±4.78	0,382

The hormone levels between deposition and the end of the CP, as well between the end of the CP and coupling, were lower than those measured between coupling and egg laying and were significantly different (P <0.01). Although estradiol levels increased before coupling, there were no significant differences between coupling and egg laying. Hormone levels increased almost 3-fold between deposition and the end of the CP, and those levels were maintained over the subsequent subperiods (Table 2).

**Table 2:** Mean FPM (ng/g) and Mean FEM (pg/g) levels during the reproductive cycle, further subdivided. The number of fecal samples obtained for each subperiod of the reproductive cycle is shown in brackets. ANOVA and Bonferroni Post Hoc Test was performed. A, B, C: P ≤0.05.

	From egg laying to CP end (5mm)		From CP end (5mm) to coupling (n = 8)	From coupling to egg laying			P
	Within one month of laying eggs (n = 6)	After at least 1 month from laying eggs (n = 8)		Follicles <20mm (n = 35)	Follicles 20-30mm (n = 9)	Follicles >30mm (n = 22)	
**FPM (ng/g)**	26,67±3.44 a	47,75±5.73 ab	66,75±4.15 abc	96,28±9.42 b	86,33±16.06 abc	110,12±11.26 bc	0,002
**FEM (pg/g)**	110,31±25.41 b	61,74±18.20 a	103,57±14.85 b	65,16±5.25 a	103,09±14.85 b	79,15±9.14 a	0,023

This subdivision had some significant variations in relation to the FEM level. In particular, a higher level of FEM appeared within one month post-deposition, compared with that of the subsequent period, which ended with a rise in room temperature to 31°C (P<0.05). Between this rise in temperature and the coupling, the FEM level increased significantly and then decreased again after coupling (P<0.05) (Table 2).

## Discussion

To improve the effectiveness of professional breeding programs, reliable techniques to monitor the animals’ gonadal function are needed [7,11]. Our study is the first one to integrate anamnestic and behavioural evaluations with the use of two non-invasive diagnostic techniques, i.e. ultrasonography and faecal sex steroids analysis.

### Ultrasound assessment

For many unconventional species including reptiles, it is important to consider the animal’s sensitivity and stress levels and choose minimally invasive procedures, particularly if frequent monitoring of healthy individuals if needed [13,76,86]. Concerns about ultrasonography may be operator-dependence and the presence of artifacts [4,18,96]. Differently to Wasmeier et al. [96], IAOV was always similar to IEOV, being numerically lower in the first interval, higher in the second and lower in the third phase. Despite the differences between the average values, not significant differences were found, probably in relation to the wide range in follicle dimensions within phases. Based on our results we can affirm that the single operator approach can be accurate in this kind of measurement. The higher variabilities found in the last phase are probably dependent on the small dimensions of the follicles inducing a higher measurement error. The presence of air trapped under the scales as well as rib shadowing, are possible ultrasonographic artifacts which could compromise an adequate highlighting of coelomic structures [4,18]. Penetration of ultrasound waves has been optimized by applying an abundant layer of ultrasound gel between the probe and the animals body. Also, considering that rib shadowing may mimic the presence of follicles if near a large vessel, it was helpful useful to use the Color Doppler [4,18]. Usually the presence of these artifacts does not prevent a clear visualization of follicular structures [4]. Also in the present study, although the artifacts mentioned have been recognized, a clear evaluation of reproductive structures has been possible. Through manual containment and a ventro-lateral approach, visualization of the python’s ovarian structures was possible, which is in accordance with the literature [18,24]. In this study a better visualization of structures was obtained by approaching on the right, except in one subject, who showed no substantial difference between sides. Currently, there is no data in the literature regarding a side preference for visualizing the tissues. Pythons have remarkable phenotypic plasticity of their visceral organs in response to ingestion of meals [97]. However, we did not notice significant displacement of the reproductive structures in relation to the time since the last meal, so as to prevent their evaluation. Follicles and eggs showed different ultrasound characteristics during the reproductive cycle, suggesting the existence of successive and distinct phases. Based on data collected from the animal’s medical history, behavioural observation and ultrasound evaluation, we identified four phases of the reproductive cycle as follows:

- Anovulatory phase, from egg laying to the end of the CP and the appearance of at least one follicle ≥5 mm in diameter
- Transition, from the end of the previous phase to coupling
- Folliculogenesis, from coupling to the ultrasound recognition of the embryo
- Embryogenesis, from the end of folliculogenesis to egg laying

We also noted an additional phase that occurs when follicular development is not complete, indicated as follicular regression. This is similar to what has been previously reported in the literature about royal pythons, regarding the distinction between “follicular quiescence, atresia, and development to oviposition” [4]. Other authors have noted the presence of pre-vitellogenic follicles, vitellogenic follicles and eggs for both royal pythons and other snake species, such as the rainbow boa (*Epicrates cenchria*) and the reticulated python (*Python reticulatus*) [7,44,98].

#### Anovulatory phase

The anovulatory phase is characterized by the absence of significant ovarian activity. Prey was consumed regularly throughout this phase, as reported in the literature [2,5], and it was possible to recognize the ovarian stroma, due to the presence of small anechoic follicles. Egg deposition in the wild occurs primarily during the second half of the dry season, i.e., from mid-February to early April, then the females go into a period of reproductive quiescence until the second rainy season, between mid-September and mid-November [2,5,99]. In captivity, these environmental stimuli are simulated through conditioning, to encourage ovarian activity and passage from the anovulatory phase to the transition phase. Animals bred in standardized conditions experience a similar duration of the anovulatory phase and a fairly homogeneous response to the artificial variation in environmental conditions (especially temperature); therefore, they typically resume follicular development. This is in line with what has been reported in the literature; several authors emphasize the importance and effectiveness of conditioning [5,6,7].

#### Transition phase

In this study, the resumption of follicular development was identified as a transition phase. Considering the average duration of this phase, variability among individuals was noted by the high standard deviation value. This is in accordance with the literature, in relation to both interspecific and intraspecific variability in reptile reproduction [5,100,101]. Therefore, despite the uniform response of the females to this conditioning, the continuation of follicular development and ovarian activity could be influenced by other factors in addition to temperature, probably linked to the female itself, though without obvious clinical alteration. These factors diversify the reproductive response of individuals reared in standardized conditions, resulting in different follicular growth rates and different durations of the transition phase. Prior to mating, the animals fed regularly, in accordance with the literature [2,5].

#### Folliculogenesis

After mating, and the start of folliculogenesis, prey was still offered but the intake was less. The females also spent more time on the colder side of the rack, as well as inside the water dish. This is in line with the data reported in the literature regarding follicular development, less food intake, and the attempt to lower their body temperature [2,5]. At this stage, the ultrasound showed that the central echogenicity of the follicles was intensifying, likely due to yolk formation. This corresponds to the literature, in that during vitellogenesis the follicles change their colour from white to yellow and increase their size by 10 to 100 times [102–104]. Additionally, during folliculogenesis, hepatomegaly was observed in some specimens. This confirms the literature in other reptile species, which reports that the liver significantly increases in volume during vitellogenesis, due to the role of the liver itself in transforming lipids into vitellogenin wich is subsequently deposited in the follicles [103,105]. Although we can not exclude that the phenomenon is related to the digestion of a meal. Ovulation occurs during the follicular phase and is described as a phenomenon accompanied by the swelling of the caudal third of the female’s body, with considerable variability among animals [2,5,6,7]. In this study, it was not possible to consistently identify the moment of ovulation; however, the pre-egg laying shed was seen about a month prior to egg deposition, confirming the literature [5]. Non-fertilized eggs, called slugs, are sometimes laid and are characterized by reduced dimensions and altered colour (yellowish) [5]. We evaluated the possibility of differentiating fertilized eggs from slugs early in the cycle, and due to the slugs’ irregular appearance, they can be recognized about three weeks prior to deposition. There is no current literature on this subject.

#### Embryogenesis

Ultrasonography to evaluate embryonic vitality has been used in some reptiles species with good results [16,18,23–25,36,42]. We evaluated the embryos in *P*. *regius*, an oviparous species. Indeed, the embryonic vesicle can be observed about two weeks before deposition. By colour Doppler, it is possible to estimate the degree of the vascularization and initial cardiac activity near deposition. In particular, the systolic and diastolic peak velocity as well as heart frequency can be highlighted. This could be an important tool for monitoring the embryo viability. In the case of the royal python, it is possible to monitor the embryo development by following the increased size and evaluating the vascularization.

#### Follicular regression

We observed cases of follicular regression and distinguished such cases from physiological atresia that affects follicles during follicular development. Physiological atresia implies the normal reduction in follicle number during the reproductive cycle, from the highest number of follicles in the beginning to the lowest number of eggs deposited [4,47,106]. We considered follicular regression as the phenomenon that involves a massive reabsorption of follicles that had already reached development, typical of the folliculogenesis, resulting in a return to the previous phases (anovulatory or transitional) until the beginning of the next cycle. This is in line with the literature. Cases of follicular regression where the reproductive cycle was completed without egg deposition have been reported in the royal python, as well as other snakes [4,8]. The causes of this phenomenon are not currently known. In the *Python brongersmai*, a connection with the presence or absence of the male at the time of follicular development was suggested [8], but has not been verified, as all reproductive females in the study were individually housed during follicular development, but not all experienced follicular regression.

#### Egg and slug counts

In this study, the average number of fertile eggs by deposition was equal to 6.3, which is consistent with the literature. In royal pythons, the average number of eggs laid is 6.5, and the average range is from 1 to 11 [6,7]. In comparing the two successive cycles, there were no significant differences in the phase duration, the follicular growth rate or the average number of fertile eggs laid. A significant increase in the average number of slugs was noted, without substantial changes in the management of the snakes. Slugs have been highlighted both in viviparous and oviparous species. To date, there are no clear explanations in the literature about their aetiology, but it seems to be linked to the presence of underlying pathologies of the females [107].

#### Hormonal trends evaluation

In addition to ultrasound evaluations, hormone tests for progesterone and 17β-estradiol were performed. To have a better picture of the female’s reproductive activity, the ultrasonography data was integrated with the data on the sex steroids [108]. Detection of sex steroid levels in the serum is the most direct method of data collection; however, blood sampling can be difficult due to venous access and animal stress. Therefore, non-invasive detection methods were used, including the analysis of faecal and urinary hormone metabolites [60,61,63–74]. However, biological and technical factors related to the sampling and analysis (such as sex, age, and reproductive status of the animal, sample storage and transportation, the representativeness of the sub-sample selected for extraction and analysis if is not possible to collect the entire faecal mass) could complicate the interpretation of these results and must be considered and possibly standardized [78,109–112]. In this study, an increase in progesterone levels was observed during the reproductive cycle and then dropped sharply at the end of embryogenesis. Progesterone levels appeared significantly higher in the period between pairing and egg laying than in the anovulatory and transition phases. Progesterone levels increase during gestation in many vertebrate species to maintain the pregnancy. Also with regard to the non-mammalian vertebrates, this hormone seems to play important reproductive roles, such as deposition of egg-white proteins and inhibition of myometrium contractility. Egg progression is also favored by progesterone, as well as eggshell formation [49,55]. In viviparous species, the level of this hormone decreases after giving birth [49]. To date, there are no studies in the literature on the level of sex steroids in the royal python, either for plasma or faeces. However, some studies have been carried out on plasma sex steroid assays in other snake species. Considering the trend in hormonal levels, there appears to be a correlation between the increased progesterone in the royal python and the levels reported in other species. The plasma progesterone level appears to be significantly higher during gestation than during follicular development, which lowers after egg deposition in different snake species of snakes including the western diamondback rattlesnake (*Crotalus atrox*), monocled cobra (*Naja kaouthia*), cascavel (*Crotalus durissus terrificus*), western garter snake (*Thamnophis elegans*) and water moccasin (*Agkistrodon piscivorus*) [49,52,53,106,113,114]. Thus, the results from our study seem to support what is reported in the literature about the likely role of this hormone in maintaining gestation among reptile species [52–55]. Progesterone trend appears to be inversely related to the number of follicles and directly proportional to the follicles size. The faecal metabolites of this hormone appear to increase after the coupling, when the number of follicles decreases and instead the size of the follicles increases. However, these hypotheses cannot be expressed for estradiol, for which no trend with statistically significant variations during the reproductive cycle has been highlighted. Indeed, in this study the trend of faecal 17β-estradiol metabolites appeared irregular, with higher values within one month after deposition, and shortly before coupling. These results, however, are not in agreement with those reported in the literature, and differ from those of the plasma hormone levels for other snake species. The rattlesnake (*Crotalus atrox*), the monocled cobra (*Naja kaouthia*) and cascavel (*Crotalus durissus terrificus*) show higher levels of estradiol during follicular development and coupling than during gestation [49,106,113]. In the western rattlesnake (*Crotalus oreganus*), no significant increase during the reproductive cycle has been reported [115]. There are currently few studies about the levels of sex steroid faecal metabolites in chelonians, veiled chameleons (*Chamaeleo calyptratus*) and blue-tongued lizards (*Tiliqua nigrolutea*), the latter was evaluated by monitoring the male reproductive activity [48,94,95]. Researchers considered the reproductive cycle of female veiled chameleons and noted a significant correlation between the plasma level concentrations of 17β-estradiol, progesterone and testosterone faecal metabolites and their respective hormones [48]. In addition, a high level of progesterone shortly before egg deposition was noted [48]. There are currently no reported studies on this method in ophidians. Though similarities between some lizards and snakes in the morphology and topography of the gonads were found. Due to the large number of species and the scarcity of available information in the literature, further studies regarding the appearance of ovarian structures during the reproductive cycle are needed [12]. Thus, it is important to carry out studies on individual species to expand our knowledge about it.

#### Study limitations

Although this study has achieved its aim, some limitations are present. First of all, hormonal profiles have not been validated in plasma measurements. This implies that we do not really know if there is a correlation between faecal and plasma hormonal levels. Moreover, having no precise numerical ranges and a sure match with plasma hormone levels, further interesting correlations can only be hypothesized, such as a correlation between hormonal levels and the size of the follicles. This limit mostly due to compliance with legislation (D.Lgs. of March 4, 2014, no. 26 which has implemented the Directive n. 2010/63 / EU), which requires that scientific studies not carried out on experimental animals kept in experimental establishments approved by the Ministry of Health, but bred for example by a private breeder (as in the case of the present study) cannot rely on techniques that cause pain equivalent to or greater than that caused by inserting a needle and therefore cannot rely on performing blood sampling. Moreover, due to the physiology of the species, in the subgroup considered for hormonal analysis every month the available samples were collected but it was not possible to have a sample for each animal at each stage of the reproductive cycle. Therefore, we got a pool of faecal samples for each reproductive stage. However, this study is intended as a descriptive evaluation of the reproduction cycle of the Royal python in the highest respect for animal welfare and hence in an absolutely noninvasive way. Thus, ss regards the hormonal evaluation, the aim of the study was not to identify numerical reference ranges, but rather only to evaluate whether for one or both of the hormones studied (progesterone and 17β-estradiol) was recognizable a trend of faecal metabolites apparently linked to the phases of the reproductive cycle, characterized by ultrasound. This evaluation has never been done before, and is just preliminary. Highlighted this apparent trend for a hormone (progesterone) and not to the other (17β-estradiol), surely we aim to deepen this topic evaluating the existence of a correlation with their plasma levels, on experimental animals, as a purpose of further study. Another limitation of the study, is related to the breeding management. After the oviposition all eggs have been removed and incubated separately; this may imply a change in hormone levels compared to those evaluated in females left with their eggs for the incubation period. However, having no control group, this study does not lend itself to a comparison in these terms.

## Conclusions

**Fig 15:**
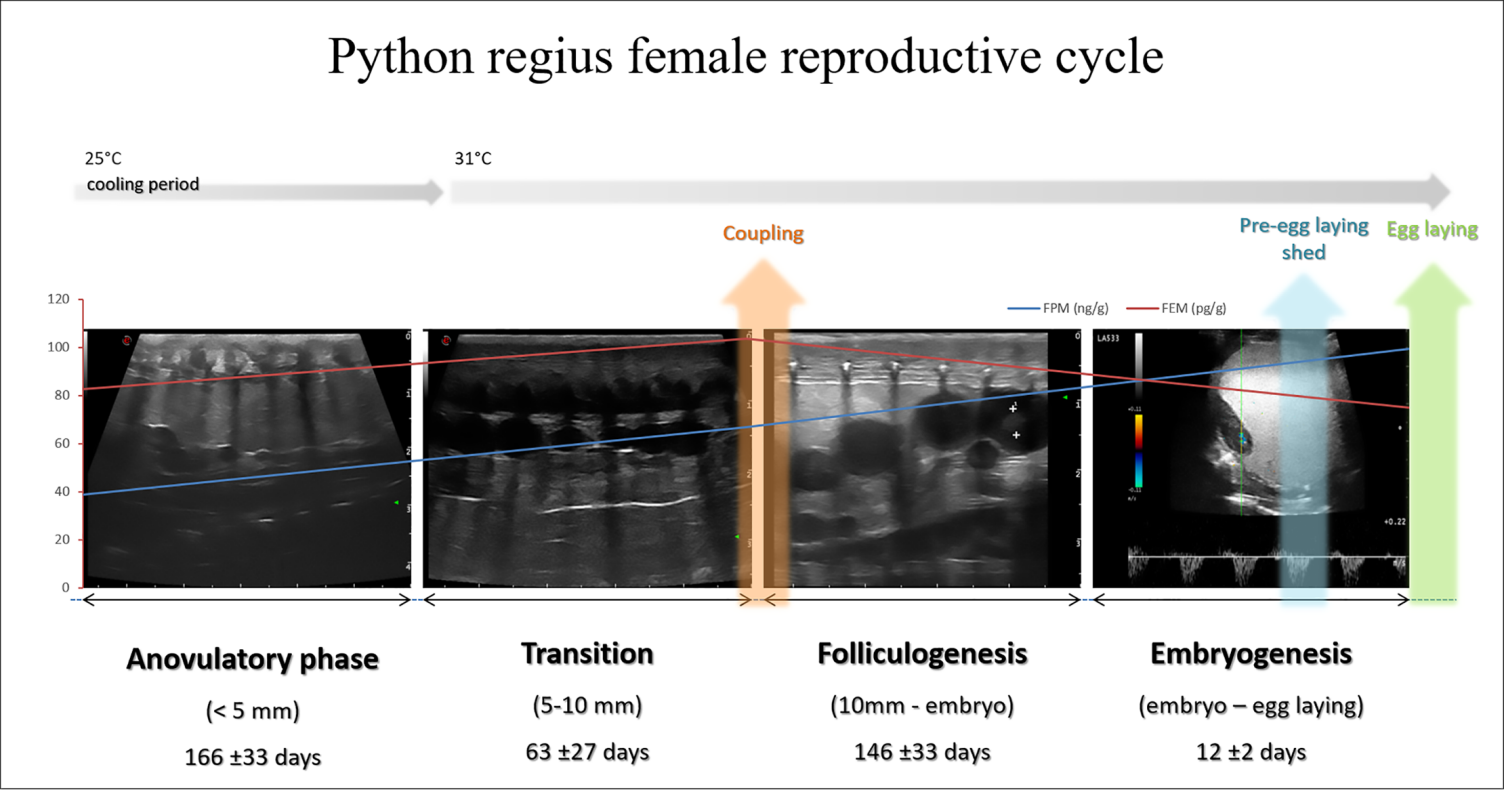
*P*. *regius* female reproductive cycle.

This study confirms previous published findings that the different phases of a female ball pythons reproductive cycle can be identified by ultrasonography [4]. This technique has also proved to be important to early recognize the presence of slugs. Furthermore, our findings highlight the utility of colour Doppler in monitoring the embryo viability, evaluating vascularization and early heart activity. This study suggests that the evaluation of sex steroid faecal metabolites levels, particularly progesterone, may provide further information on the reproductive cycle of females of this species. We believe that the female reproductive cycle of royal pythons kept in captivity can be schematized as shown in Fig 15.

In conclusion, the association between ultrasonography and the analysis of sex hormone faecal metabolites allows us to monitor the reproductive activity of female royal pythons bred in captivity.

## Supporting information

S1 Video One day before deposition. Evaluation of initial cardiac activity. B-mode video with pulsed-wave (PW) Doppler.
(AVI)

S2 Video Evaluation of initial cardiac activity by colour doppler. B-mode video with Colour Doppler, one day before deposition.
(AVI)
